# Supplementary material for: Modular co-evolution of metabolic networks
Source: BMC Bioinformatics. 2007 Aug 27;8:311. doi: 10.1186/1471-2105-8-311 (PMC2001200; doi:10.1186/1471-2105-8-311)
Supplement: Additional File 1 — Addition File for "Modular co-evolution of metabolic networks. Supplementary material for this paper [file 1471-2105-8-311-S1.pdf]

Addition File for “Modular co-evolution of metabolic networks”

1. Reference organisms

Table A1 List of the 115 reference organisms applied in the computation of phylogenic profiles of enzymes in the *H. sapiens* metabolic network

Category	Taxonomy	Organism		
Eukaryotes	1	1	hsa	*Homo sapiens* (human)
	2	2	ptr	*Pan troglodytes* (chimpanzee)
	3	3	mmu	*Mus musculus* (mouse)
		4	rno	*Rattus norvegicus* (rat)
	4	5	cfa	*Canis familiaris* (dog)
	5	6	xla	*Xenopus laevis* (African clawed frog)
		7	xtr	*Xenopus tropicalis* (western clawed frog)
	6	8	dre	*Danio rerio* (zebrafish)
	7	9	dme	*Drosophila melanogaster* (fruit fly)
	8	10	cel	*Caenorhabditis elegans* (nematode)
	9	11	ath	*Arabidopsis thaliana* (thale cress)
	10	12	sce	*Saccharomyces cerevisiae*
	11	13	spo	*Schizosaccharomyces pombe*
	12	14	pfa	*Plasmodium falciparum*
	13	15	tbr	*Trypanosoma brucei*
	14	16	ehi	*Entamoeba histolytica*
Bacteria	15	17	eco	*Escherichia coli* K-12 MG1655
		18	ecj	*Escherichia coli* K-12 W3110
		19	ece	*Escherichia coli* O157 EDL933
		20	ecs	*Escherichia coli* O157 Sakai
		21	ecc	*Escherichia coli* CFT073
		22	sty	*Salmonella typhi*
		23	stm	*Salmonella typhimurium*
		24	ype	*Yersinia pestis CO92*
		25	ypk	*Yersinia pestis* KIM
		26	sfl	*Shigella flexneri*
		27	buc	*Buchnera sp.* APS
		28	bas	*Buchnera aphidicola Sg*
	16	29	hin	*Haemophilus influenzae*
		30	pmu	*Pasteurella multocida*
		31	xfa	*Xylella fastidiosa*
		32	xcc	*Xanthomonas campestris*
		33	xac	*Xanthomonas axonopodis*
	17	34	vch	*Vibrio cholerae*
	18	35	pae	*Pseudomonas aeruginosa*

	19	36	son	*Shewanella oneidensis*
	20	37	nme	*Neisseria meningitidis* MC58 (serogroup B)
		38	nma	*Neisseria meningitidis* Z2491 (serogroup A)
	21	39	rso	*Ralstonia solanacearum*
	22	40	hpy	*Helicobacter pylori* 26695
		41	hpj	*Helicobacter pylori* J99
	23	42	cje	*Campylobacter jejuni*
	24	43	rpr	*Rickettsia prowazekii*
		44	rco	*Rickettsia conorii*
	25	45	mlo	*Mesorhizobium loti*
		46	sme	*Sinorhizobium meliloti*
		47	atu	*Agrobacterium tumefaciens* C58 (UWash/Dupont)
		48	atc	*Agrobacterium tumefaciens* C58 (Cereon)
		49	bme	*Brucella melitensis*
		50	bms	*Brucella suis*
		51	bja	*Bradyrhizobium japonicum*
	26	52	ccr	*Caulobacter crescentus*
	27	53	bsu	*Bacillus subtilis*
		54	bha	*Bacillus halodurans*
		55	oih	*Oceanobacillus iheyensis*
		56	sau	*Staphylococcus aureus* N315 (MRSA)
		57	sav	*Staphylococcus aureus* Mu50 (VRSA)
		58	sam	*Staphylococcus aureus* MW2
		59	lmo	*Listeria monocytogenes*
		60	lin	*Listeria innocua*
	28	61	lla	*Lactococcus lactis*
		62	spy	*Streptococcus pyogenes* SF370 (serotype M1)
		63	spm	*Streptococcus pyogenes* MGAS8232 (serotype M18)
		64	spg	*Streptococcus pyogenes* MGAS315 (serotype M3)
		65	spn	*Streptococcus pneumoniae* TIGR4
		66	spr	*Streptococcus pneumoniae* R6
		67	sag	*Streptococcus agalactiae* 2603
		68	san	*Streptococcus agalactiae* NEM316
		69	smu	*Streptococcus mutans*
	29	70	cac	*Clostridium acetobutylicum*
		71	cpe	*Clostridium perfringens*
	30	72	tte	*Thermoanaerobacter tengcongensis*
	31	73	mge	*Mycoplasma genitalium*
		74	mpn	*Mycoplasma pneumoniae*

		75	mpu	*Mycoplasma pulmonis*
		76	uur	*Ureaplasma urealyticum*
	32	77	mtu	*Mycobacterium tuberculosis* H37Rv (lab strain)
		78	mtc	*Mycobacterium tuberculosis* CDC1551
		79	mle	*Mycobacterium leprae*
		80	cgl	*Corynebacterium glutamicum*
		81	cef	*Corynebacterium efficiens*
		82	sco	*Streptomyces coelicolor*
	33	83	blo	*Bifidobacterium longum*
	34	84	fnu	*Fusobacterium nucleatum*
	35	85	ctr	*Chlamydia trachomatis*
		86	cmu	*Chlamydia muridarum*
		87	cpn	*Chlamydophila pneumoniae* CWL029
		88	cpa	*Chlamydophila pneumoniae* AR39
		89	cpj	*Chlamydophila pneumoniae* J138
	36	90	bbu	*Borrelia burgdorferi*
		91	tpa	*Treponema pallidum*
		92	lil	*Leptospira interrogans*
	37	93	syn	*Synechocystis sp.* PCC6803
		94	tel	Thermosynechococcus elongatus
	38	95	ana	*Anabaena sp.* PCC7120 (Nostoc sp. PCC7120)
	39	96	cte	*Chlorobium tepidum*
	40	97	dra	*Deinococcus radiodurans*
	41	98	aae	*Aquifex aeolicus*
	42	99	tma	*Thermotoga maritima*
Archaea	43	100	mja	*Methanococcus jannaschii*
	44	101	mac	*Methanosarcina acetivorans*
		102	mma	*Methanosarcina mazei*
	45	103	mth	*Methanobacterium thermoautotrophicum*
	46	104	mka	*Methanopyrus kandleri*
	47	105	afu	*Archaeoglobus fulgidus*
	48	106	hal	*Halobacterium sp.* NRC-1
	49	107	tac	*Thermoplasma acidophilum*
		108	tvo	*Thermoplasma volcanium*
	50	109	pho	*Pyrococcus horikoshii*
		110	pab	*Pyrococcus abyssi*
		111	pfu	*Pyrococcus furiosus*
	51	112	ape	*Aeropyrum pernix*
	52	113	sso	*Sulfolobus solfataricus*
	53	114	sto	*Sulfolobus tokodaii*

	54	115	pai	*Pyrobaculum aerophilum*

2. Comparison the *H. sapiens* metabolic network with its random counterparts

2.1 topological null model

Table A2 Inter-module degrees and sizes of topological modules for the randomized version of the *H. Sapiens* network shown in Figure 6

Module	Inter-module degree	number of nodes
14	3	27
25	3	16
7	4	45
13	7	33
22	8	35
16	11	32
20	11	33
19	14	28
9	18	39
15	20	29
5	21	36
23	22	20
8	25	34
4	26	40
10	27	46
1	28	44
21	29	36
2	30	37
11	32	39
17	33	43
18	34	37
12	39	44
24	41	51
6	52	60
3	56	64

2.2 biological null model

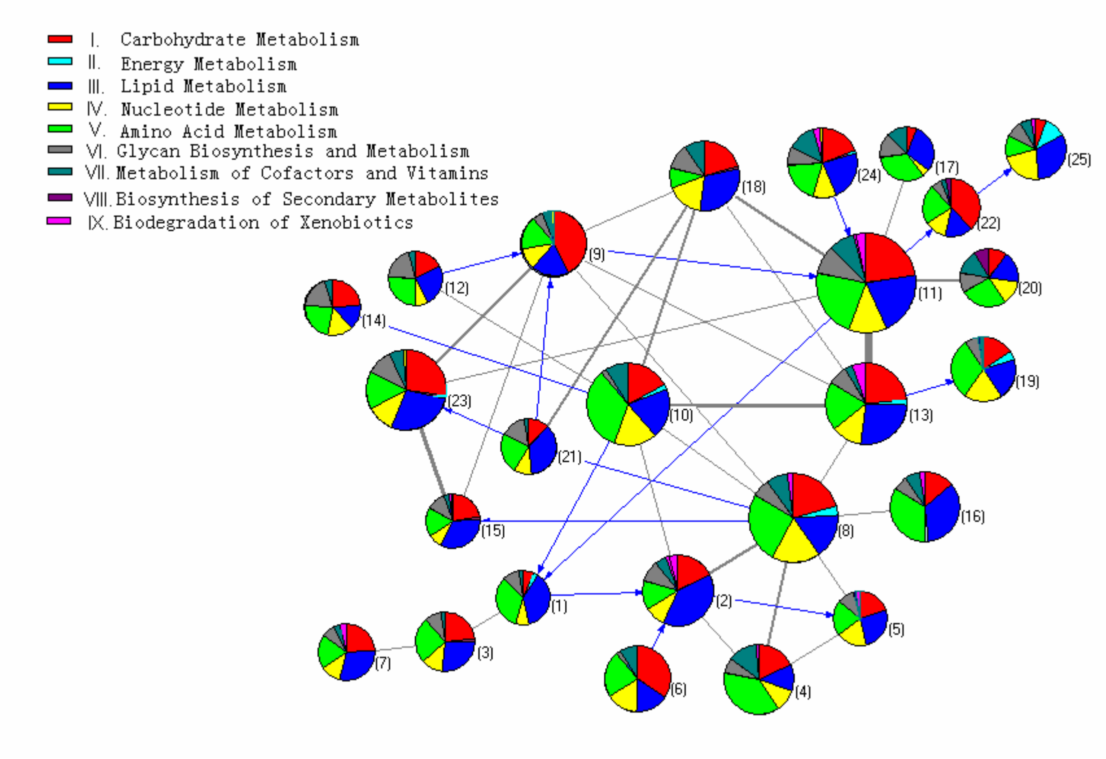

Figure A1 - Cartographic representation of the metabolic network for *H. sapiens*, in which enzymes were randomly shuffled. The topology of the network is unchanged compared with the *Homo sapiens* network, but the reactions are coupled with different enzymes.

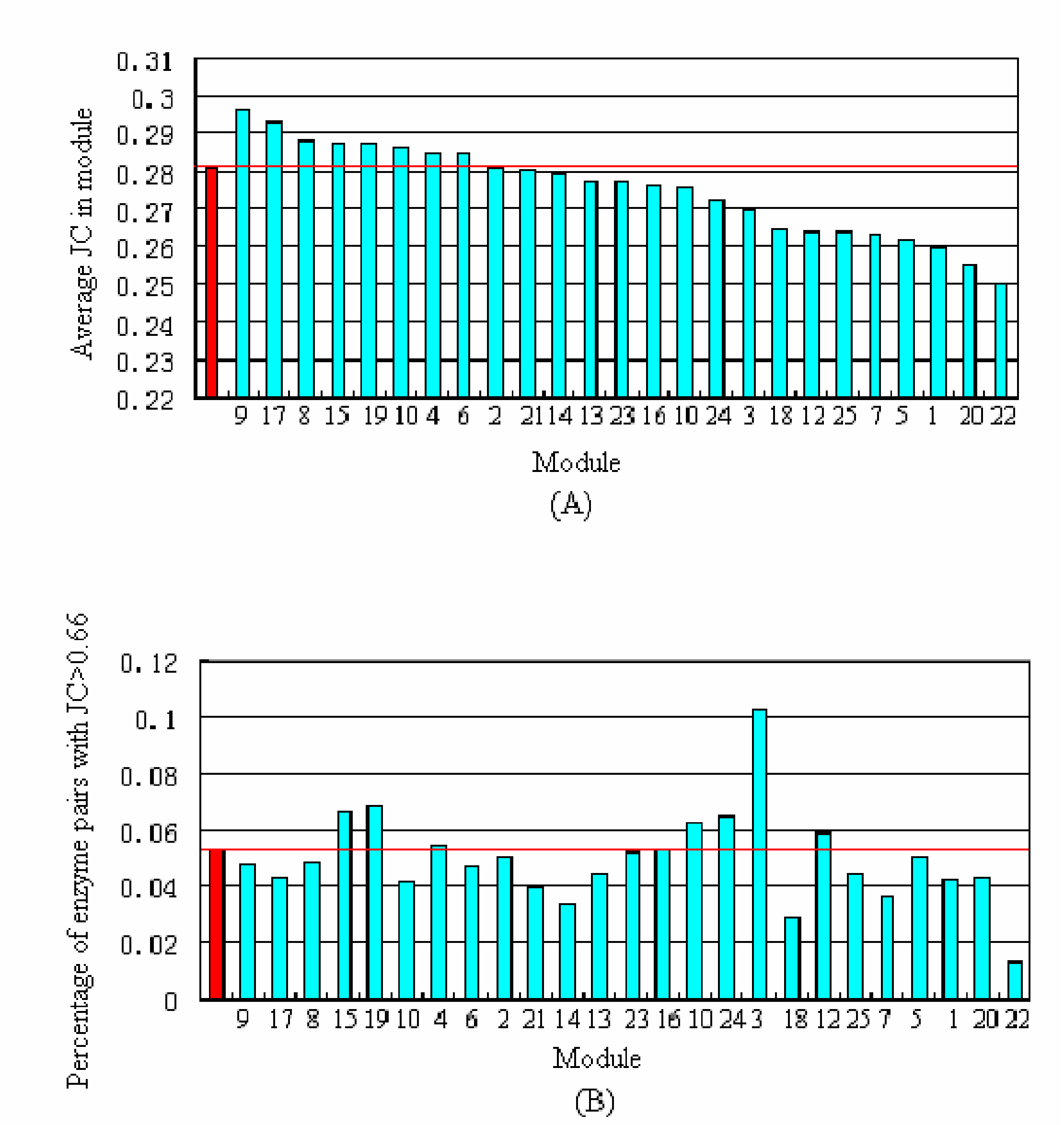

Figure A2 Comparison of the similar extent of phylogenetic profiles for enzymes pairs within each module with that within the global random network.

(A) Average Jaccard coefficient (JC) of enzyme pairs within modules.

The red column represents the global network. The modules are ordered according to their average JC in a decreasing way.

(B) Percentage of enzyme pairs within modules with JC>=0.66 (threshold definition).

The red column represents the global network. The modules are drawn in the same order as in (A).
